# Infection with *Batrachochytrium dendrobatidis* lowers heat tolerance of tadpole hosts and cannot be cleared by brief exposure to CTmax

**DOI:** 10.1371/journal.pone.0216090

**Published:** 2019-04-29

**Authors:** Andrés Fernández-Loras, Luz Boyero, Francisco Correa-Araneda, Miguel Tejedo, Attila Hettyey, Jaime Bosch

**Affiliations:** 1 Museo Nacional de Ciencias Naturales-CSIC, Madrid, Spain; 2 Department of Plant Biology and Ecology, Faculty of Science and Technology, University of the Basque Country (UPV/EHU), Leioa, Spain; 3 IKERBASQUE, Basque Foundation for Science, Bilbao, Spain; 4 Unidad de Cambio Climático y Medio Ambiente, Instituto de Estudios del Hábitat (IEH), Facultad de Arquitectura y Construcción, Universidad Autónoma de Chile, Temuco, Chile; 5 Núcleo del Estudio en Ciencias Ambientales (NEA) y Departamento de Ciencias Ambientales (Facultad de Recursos Naturales), Universidad Católica de Temuco, Temuco, Chile; 6 Department of Evolutionary Ecology, Estación Biológica de Doñana-CSIC, Américo Vespucio s/n, Sevilla, Spain; 7 Lendület Evolutionary Ecology Research Group, Plant Protection Institute, Centre for Agricultural Research, Hungarian Academy of Sciences, Budapest, Hungary; 8 Department of Ecology, Institute for Biology, University of Veterinary Medicine, Budapest, Hungary; 9 Centro de Investigación, Seguimiento y Evaluación, Parque Nacional de la Sierra de Guadarrama, Rascafría, Spain; 10 Research Unit of Biodiversity (CSIC, UO, PA), Oviedo University—Campus Mieres, Spain; University at Albany, SUNY, UNITED STATES

## Abstract

Climate change and infectious disease by the chytrid fungus *Batrachochytrium dendrobatidis* (*Bd*) are major drivers of amphibian extinctions, but the potential interactions of these two factors are not fully understood. Temperature is known to influence (1) the infectivity, pathogenicity and virulence of *Bd*; (2) host-parasite dynamics, especially when both hosts and parasites are ectothermic organisms exhibiting thermal sensitivities that may or may not differ; and (3) amphibian vulnerability to extinction depending on their heat tolerance, which may decrease with infection. Thus, in a global warming scenario, with rising temperatures and more frequent and extreme weather events, amphibians infected by *Bd* could be expected to be more vulnerable if temperatures approach their critical thermal maximum (CTmax). However, it is also possible that predicted high temperatures could clear the *Bd* infection, thus enhancing amphibian survival. We tested these hypotheses by measuring CTmax values of *Bd*-infected and *Bd*-free aquatic tadpoles and terrestrial toadlets/juveniles of the common midwife toad (*Alytes obstetricans*) and examining whether exposure of *A*. *obstetricans* individuals to peak temperatures reaching their CTmax clears them from *Bd* infection. We show that (1) *Bd* has a wide thermal tolerance range; (2) *Bd* is capable of altering the thermal physiology of *A*. *obstetricans*, which is stage-dependent, lowering CTmax in tadpoles but not in toadlets; and (3) *Bd* infection is not cleared after exposure of tadpoles or toadlets to CTmax. Living under climatic change with rising temperatures, the effect of *Bd* infection might tip the balance and lead some already threatened amphibian communities towards extinction.

## Introduction

*Batrachochytrium dendrobatidis* (*Bd*), a pathogenic chytrid fungus causing chytridiomycosis in many amphibians, is considered the most deadly invasive species on the planet [1] and a main driver behind amphibian species extinctions globally [2, 3], with profound effects on communities and ecosystems [4]. Environmental conditions can have a large influence on *Bd* host-parasite dynamics [5, 6], temperature being a major factor influencing its prevalence (i.e., the proportion of infected animals) and virulence [7]. Growth and reproductive characteristics of *Bd* are known to highly depend on temperature [8–10], and there is a negative correlation between temperature and *Bd* prevalence or pathogen load, chytrid infections often being more severe in winter or in colder areas [6, 11, 12].

Temperature can influence amphibian population dynamics through its effects on physiology of both the host and the fungal pathogen [13]. Amphibians being ectothermic, their immune system and its responses against pathogens are influenced by environmental temperature [14–17]. Fever, a crucial response to infection that has evolved both in endotherms and ectotherms, can confer a survival advantage upon infection by stimulating the immune system [18]. Infected ectotherms have been shown to raise their body temperature by seeking warmer sites and spending more time in those sites than uninfected individuals, a phenomenon called behavioral fever [19, 20, 21]. Also, several studies have reported on the utility of using elevated temperatures as a method of *Bd* elimination [22–24].

The recently proposed thermal mismatch hypothesis suggests that infection risk will decrease as the difference in thermal tolerance of host and pathogen (tolerance mismatch) increases [25]. A refinement of the hypothesis suggests that infectious disease outbreaks are most likely to occur at temperatures where the performance gap between pathogen and host is greatest in favor of the pathogen [10]. Because parasites are thermal generalists, which often have broader thermal performance breadths than their hosts, and assuming that both hosts and parasites are locally adapted to climatic conditions in their ranges, this hypothesis posits that hosts adapted to cooler climates should be especially susceptible to disease under unusually warm conditions, whereas warm adapted hosts are more prone to infection under cooler conditions (Fig 1 in [10]).

Extreme weather events have increased in intensity, frequency and unpredictability [26–30], and temperature extremes have undergone systematic and significant changes over the last decades [31]. These forms of global climate change are predicted to be more severe at higher latitudes and altitudes and over land, where they are especially likely to cause a series of malign effects, including thermal stress in many species [32] and an increase in the frequency of infectious disease outbreaks [10], leading to profound changes in ecosystem structure and function [33], and ultimately threatening the integrity of ecosystems [34]. Ectotherms are considered especially vulnerable to climate change due to the direct dependence of their fitness on temperature [35, 36], and because new daily, seasonal, or intermittent temperature cycles will most likely be shifted away from their optimum and closer to lethal extremes [37, 38].

Estimating potential risks of species and populations posed by climate change includes the assessment of the heat tolerance, such as the critical thermal maximum (CTmax). CTmax is usually quantified under controlled conditions using the Hutchison´s dynamic method, [39] in which organisms are exposed to a constant heating rate until an end-point is attained. This end-point represents the upper limit of the ability of animals to counterbalance temperature increase and marks the loss of homeostasis. According to Huey et al. [40], vulnerability of a species to rising environmental temperature depends on several factors including the species’ sensitivity to temperature change, its capacity to adapt to such change, its resilience, and its exposure level. Some studies have proposed that tropical ectotherms will be more susceptible to warming-induced extinctions than their temperate counterparts, since their CTmax is only slightly higher than the highest ambient temperatures they already experience, which leaves these species with an extremely low warming tolerance [40, 41]. Importantly, infection with *Bd* may result in reduced CTmax, as shown for the tropical anuran *Litoria spenceri* at the adult stage [42]. Previous studies indicated that *Bd* infection correlates with cooler temperatures in the field [11], and laboratory experiments have demonstrated that *Bd* ceases growth at temperatures above 28°C [8]. Then, the predicted increase in temperatures may indirectly determine a protection of amphibians from *Bd* and an experimental heating of hosts may clear themselves of the pathogen.

Within this context, we conducted a thermal ramping experiment [43] in both aquatic tadpoles and recently metamorphosed, terrestrial toadlets of two contrasting altitude populations of *Alytes obstetricans*, an anuran species from temperate Europe which has been hit hard by chytridiomycosis [44, 45]. Our goals were (1) to determine if gradually and briefly elevating environmental temperature close to the CTmax of individuals can be used to clear *Bd* infection from both host stages–an experimental heating procedure similar to heating pulses employed to trigger cleaning *Bd* from amphibian hosts [42]–and (2) to assess whether infection with *Bd* lowers upper thermal tolerance limits of tadpoles or toadlets in this species (as suggested by reference 42).

## Material and methods

We collected a total of 121 *A*. *obstetricans* specimens, both larval (80) and recently metamorphosed, toadlets (41) from two localities: Toro, at mid-altitude population (Zamora, Central Spain, geographic coordinates: 41.37 N, 5.44 W; altitude: 740 m above sea level; 80 tadpoles and 21 toadlets collected) and Acherito, a montane population (Huesca, Northern Spain, geographic coordinates: 42.88 N, 0.71 W; altitude: 1875 m above sea level; 18 tadpoles and 20 toadlets collected). Prevalence of *Bd* infection in larval stages is known to approach 100% during colder months at both localities [11, 45]. Animals were collected in November 2012 and May 2013 at Toro and in August 2013 at Acherito.

Prior to the heating experiment, we assessed the developmental stage of tadpoles and measured body mass of tadpoles and toadlets. Tadpoles originating from Toro were between developmental stage 26 and 37 [46] and weighed 1.05 ± 0.03 g (mean ± SE), while those originating from Acherito were between stage 34 and 40 and weighed 1.56 ± 0.07 g. Toadlets collected from Toro weighed 0.65 ± 0.03 g and those collected from Acherito weighed 1.96 ± 0.07 g. We acclimated animals for 2 days in 1-L individual containers at 18°C and a light:dark cycle of 12:12. Containers were filled with dechlorinated tap water in the case of tadpoles, while containers with toadlets were lined with sterilized paper towels moistened with dechlorinated tap water. Every 72 hours we changed water and replaced the wet paper towels, and subsequently fed tadpoles and toadlets ad libitum with commercial food for amphibian tadpoles or with baby crickets, respectively.

In order to determine the CTmax (°C) of tadpoles and toadlets, we individually placed animals within a new plastic container and subjected them to a thermostated bath (HUBER K15-cc-NR). Initial temperature was 20°C in the case of tadpoles and 19.8°C for toadlets. Following Hutchinson’s dynamic method for determination of CTmax [47], we increased water temperature at a constant rate of 0.8°C min^-1^ and observed tadpoles and toadlets continuously until they reached the endpoint. We defined the endpoint as the point at which tested individuals become motionless and fail to respond to external stimuli by prodding 10 consecutive hits applied each 2 s with a wooden stick [48]. We established a humane endpoint when animals did not recover motion after 30 min of reaching their CTmax. If so, animals should be euthanized with an overdose of tricaine methanesulfonate (MS222, Sigma-Aldrich, Saint Louis, MO, USA) buffered with NaHCO_3_ (no animal reached the humane endpoint criteria). Twenty tadpoles and five toadlets were assigned to the control groups and were subjected to the bath but not to heating.

In order to examine whether heating to CTmax of the host promotes a cleaning of *Bd* infection, immediately before assays, the keratinized mouthparts of tadpoles and the lower ventral surface and hind limbs of toadlets were swabbed using sterile cotton-tipped swabs (MW100-100; Medical Wire & Equipment Co, Corsham, UK). After heating experiments, tadpoles and toadlets were individually kept for an additional 15-day period in new 1-L containers with dechlorinated tap water or sterilized moistened paper lining, respectively, to allow enough time for *Bd* DNA from dead chytrid cells to degrade. Tadpoles were then euthanized with an overdose of tricaine methanesulfonate buffered with NaHCO_3_, and whole tadpole mouthparts were collected and fixed. Surviving toadlets were swabbed on day 15 and released at the exact point of capture, whereas animals found dead upon daily screenings were toe-clipped immediately. Tissue samples were stored in 70% ethanol and swabs were stored dry at 4°C until processing.

The experiments here performed were carried out in accordance with all current European directives and Spanish laws, and approved by the competent authorities of the Consejería de Medio Ambiente from Junta de Andalucía (Ref. 12_44). Procedures conformed to the recommended guidelines or use of live amphibians and reptiles in laboratory research (ASIH 2004). All experimental protocols were approved by the ‘Comité de Ética de Experimentación Animal CEEA-EBD’. All researchers implied in the experiments (AFL, JB, MT) have the competent accreditation (Category C) according to the EU Directive 2010/63/EU Article 23.2 accredited by the Federation of European Laboratory Animal Science Associations (FELASA).

We extracted *Bd*-DNA using PrepMan Ultra Reagent Protocol as described by Boyle et al. [49]. Extracted DNA was stored at -20°C until further processing. We assessed the burden of infection using the quantitative PCR (qPCR) protocol described by Boyle et al. [49] with a CFX96 thermocycler (Bio-Rad). Each plate included samples, a negative control and four standards ranging in concentration from 100 to 0.1 *Bd* zoospores ml^-1^ genome equivalents (GE), all in duplicate (isolate IA042 from Acherito). Samples were scored positive when both replicates received GE-estimates ≥ 0.1 and amplification curves had the typical sigmoidal shape.

Infection loads for tadpoles and toadlets before and after thermal treatments were compared using linear mixed models, with individuals as a random factor, and time (before/after), population, treatment (control/heated), and the interactions time x population and time x treatment as fixed factors. Infection load was transformed (log_10_) to reach normality but also to reduce differences between values obtained by swabbing the oral discs (initial) and by using the whole oral disc (final). We analysed variation in Box-Cox transformed values of CTmax of tadpoles and toadlets separately using general linear models (GLM) because all tadpoles collected from Acherito and no toadlets collected from Toro were infected. We entered population origin as a fixed factor and *Bd* infection load and body mass as covariates. JMP Pro 12 (SAS Institute Inc., NC, USA) was used for all statistical analyses.

## Results

### Initial prevalence and infection load

Initial prevalence of infection in tadpoles originating from Toro was 50% (n = 80) whereas it was 100% (n = 18) in tadpoles taken from the Acherito population. Similarly, none of the 21 toadlets collected at Toro were infected, while most toadlets collected at Acherito were infected (prevalence: 90%, n = 20). *Bd* loads of infected tadpoles from Toro assigned to the experimental group ranged from 960 to 12970 GE (average 3846 GE), whereas infected tadpoles assigned to the control group ranged from 930 to 1440 GE (average 4419 GE). Tadpoles from Acherito presented an averaged *Bd* load of 511 GE, ranging from 6 to 2330 GE. Infected toadlets from Acherito assigned to the experimental group ranged from 1 to 2690 GE (average 472 GE), while infected ones assigned to the control group ranged from 7 to 71 GE (average 115 GE).

### Effect of host heating to CTmax on *Bd* prevalence and infection load

**Fig 1:**
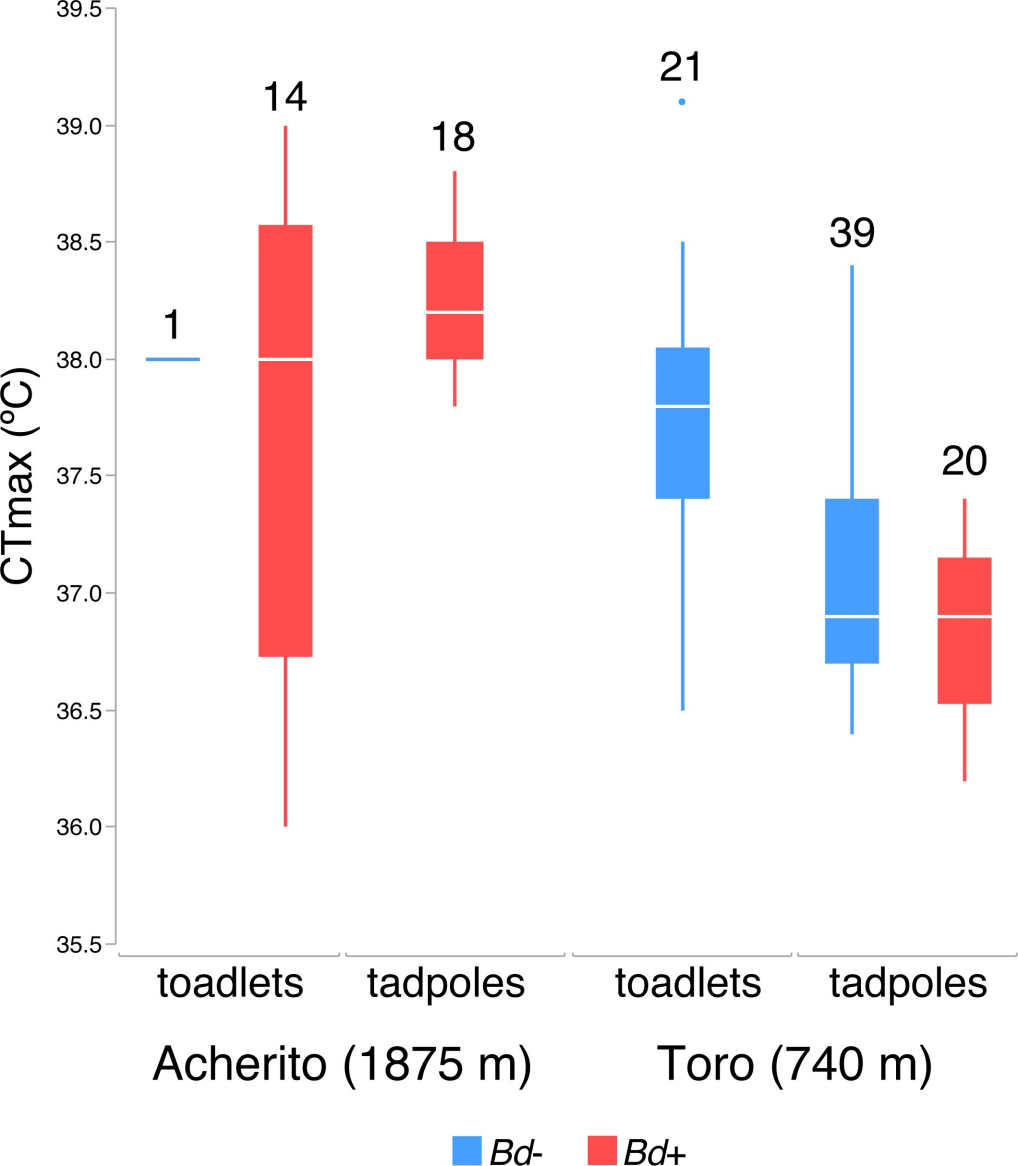
CTmax (°C) in both tadpoles and toadlets from the two studied localities, Acherito and Toro. Uninfected animals appear in blue and *Bd*-infected animals are in red. Numbers above each box-plot are sample sizes. Horizontal lines depict medians, boxes represent interquartile ranges, whiskers extend to minima-maxima, dots show potential outliers.

All tested tadpoles (78) and toadlets (36) recovered after reaching their CTmax. Averaged CTmax values were very high and extremely similar for both tadpoles (37.3°C) and toadlets (37.7°C; Fig 1). Heating to CTmax did not clear *Bd* infection. None of the 20 tadpoles collected from Toro, neither the 18 tadpoles from Acherito that tested positive for *Bd* at the start, changed their infection score. Similarly, all 20 control tadpoles maintained their infection status after completing the experiment.

**Fig 2:**
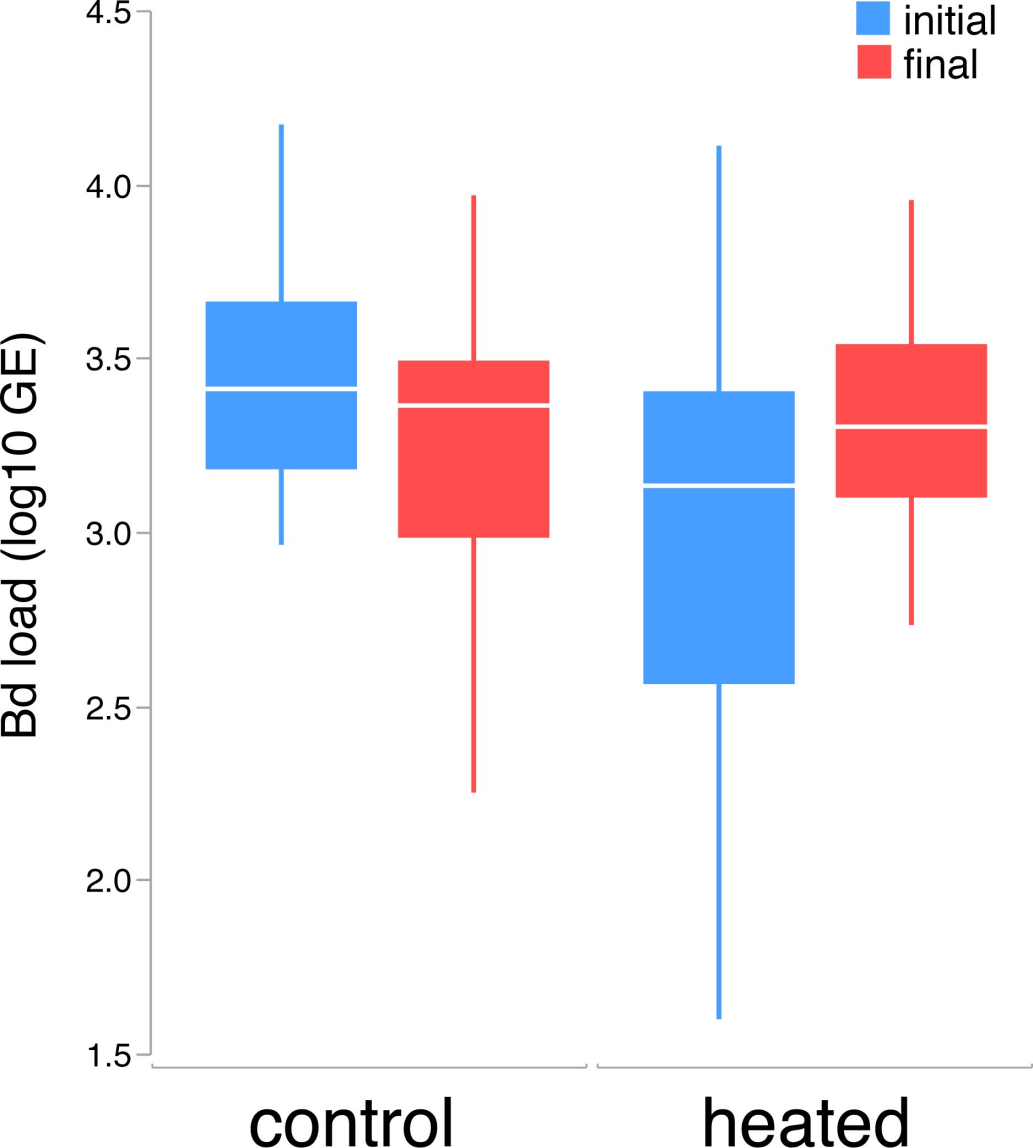
*Bd* loads (GE, log10 transformed) in both groups of tadpoles (control and heated) at the initial and final time. Horizontal lines depict medians, boxes represent interquartile ranges, whiskers extend to minima-maxima.

Larval populations differed in *Bd* loads (F_1,103_ = 23.699, p < 0.001), which were higher in Acherito. Although no significant differences were found between initial and final larval *Bd* loads (F_1,80_ = 0.970, p = 0.328) or between control and heated tadpoles (F_1,80_ = 0.001, p = 0.973), the interaction between these two factors was significant (F_1,80_ = 8.837, p = 0.004), indicating that heated tadpoles, but not control tadpoles, increased their infection load after the experiment (Fig 2).

All toadlets from Toro survived until completion of the experiment, 15 days after exposure to CTmax. On the other hand, only four of the 20 toadlets collected at Acherito survived until the end of the experiment. From those four toadlets, two were *Bd*-free and the other two had low *Bd* loads (9 and 27 *Bd* zoospore GE, respectively) at the beginning of the experiment. Most toadlets died 6 days (13 animals) after the beginning of the experiment, the rest died on day 9 (3 animals). Toadlets that died on days 6 and 9 did not differ in *Bd* loads at the beginning of the experiment or on the day of death (Student’s t-tests; t<0.8, p<0.43 in both cases). None of the 14 Acherito toadlets that were initially *Bd*-infected lost their infection after going through the CTmax experiment.

Toadlets showed no significant differences between initial and final *Bd* loads (F_1,15_ = 0.454, p = 0.511) or between heated and control animals (F_1,15_ = 1.764, p = 0.204), and the interaction between these two factors was not significant (F_1,15_ = 0.004, p = 0.951).

### Effect of *Bd* infection load on thermal tolerance limits

The linear model used to analyse differences in CTmax of tadpoles was highly significant (R^2^ = 0.63, F_3,76_ = 41.359, p < 0.001). We observed a significant difference in CTmax between the two studied populations (F_1,76_ = 61.28, p < 0.0001), with CTmax being higher in tadpoles collected from Acherito than those obtained from Toro (mean ± SE: 38.238 ± 0.075 vs. 36.975 ± 0.061°C). Most importantly, a significant negative relationship between *Bd* infection load and CTmax was found (F_1,76_ = 5.77, p = 0.0189), with a slightly lower CTmax in infected (37.046 ± 0.083°C) than non-infected tadpoles (37.500 ± 0.127°C). The effect of larval body mass had a marginally non-significant influence on CTmax (F_1,76_ = 3.45, p = 0.0673). In the case of toadlets, neither population origin nor body mass or *Bd* infection status had a significant effect on CTmax values (R^2^ = 0.03, F_3,35_ = 0.3238, p = 0.8081).

## Discussion

Our main observation that CTmax values obtained for *Bd*-infected tadpoles were significantly lower than those of uninfected ones supports similar results reported for the adult stage of the Australian frog *Litoria spenceri* [42]. Therefore, this fungal pathogen may be capable of altering the thermal physiology of the hosts it infects, or, in a narrower sense, to lower their ability to withstand high temperatures. In a global warming scenario this could have serious conservation implications for many amphibian species, especially for tropical species, which often live close to their thermal limits [41, 48]. On the other hand, temperate amphibian species may be relatively secure from similar impacts of warming, since their warming tolerance (the difference between their CTmax and environmental temperatures) is higher in most cases [41]. Because permanent ponds are in general deeper and cooler than shallow ephemeral water bodies, *A*. *obstetricans* and many other species using permanent ponds as their larval habitat could be on the safe side in this respect, while species spawning and developing in temporary water bodies may be exposed to higher risk. Nonetheless, amphibians of the temperate zone may also be highly vulnerable to climate change, because temperatures are predicted to rise more steeply in these regions [50], and, coupled with the observation of *Bd*-infection lowering CTmax, the presence of the chytrid fungus may push local populations towards extinction. However, we have to note that the decrease in CTmax accountable to *Bd*-infection was less than 0.5°C for tadpoles and a similar effect could not be detected in toadlets, while CTmax was still higher than 37°C in infected tadpoles and even higher in toadlets. Thus, our results indicate that *Bd*-infection may lower upper thermal tolerance limits of amphibians, but this decrease is minimal in *A*. *obstetricans* and will have to be assessed in a variety of other species before we can determine the importance of this effect.

It is generally believed that temperature tolerance of *Bd* ranges from 4 to 25°C, with its thermal optimum for growth and reproduction falling between 17–25°C [8–10]. However, *A*. *obstetricans* optimum thermal breadth (TB80) for larval growth was much warmer, ranging between 21–28°C (M. Tejedo, P. Pintanel, unpublished results), thus suggesting the prediction of thermal mismatch hypothesis [10]. In our experiment, where we exposed infected tadpoles and toadlets to elevated temperatures, we did not observe clearance of infection even though the CTmax, and thereby the highest ambient temperatures reached around 37.5°C, which is almost 10°C higher than the CTmax of *Bd*. An *in vitro* study [51] showed *Bd*-wipeout in all cultures after only 4 hours of exposure to 37°C. We know of only two studies that exposed amphibians to similarly high temperatures in order to clear *Bd*-infection: Woodhams and colleagues [22] exposed juvenile frogs to 37°C for 8 hours on two consecutive days, which resulted in clearance of infection in all individuals; in the other study, exposure to 35°C for one day, preceded by 30°C for 12 hours, was ineffective in clearing *Bd* from adult frogs [52]. In our experiment, we elevated temperature from 20°C to around 37.5°C at a rate of 0.8°C min^-1^, meaning that infected animals spent ca. 12 min at temperatures beyond 28°C, the upper thermal limit of *Bd*, and 4 min at temperatures beyond 35°C, which is likely too brief to kill *Bd* or alternatively, triggering host immunity [53]. From these studies it appears that even temperatures close to the thermal maximum that amphibians can withstand without lasting damage have to be maintained for more than just a few minutes. The effective combinations of elevated temperatures and duration of application/exposure of thermal stress in order to clear *Bd*-infection or at least to largely suppress infection loads remain to be determined.

To conclude, our study shows that besides other malign effects on its amphibian hosts, *Bd* can also reduce their critical thermal maximum (CTmax), at least for tadpoles. A reduction in thermal tolerance can have serious consequences for the persistence of amphibian populations at many localities worldwide, especially under the ongoing process of global climate change. At the same time, our results and those of previous studies suggest that short spikes in peak temperatures are unlikely to clear *Bd*-infection from amphibian hosts. Finally, our study draws attention to the importance of determining effective combinations of time and temperature parameters in order to deploy optimized and safely applicable disinfection treatments against this deadly disease.

## Supporting information

S1 Dataset From experimental trials examining CTmax of both tadpoles and toadlets from the two studied localities. (XLS)
